# Comparing the Similarity of Responses Received from Studies in Amazon’s Mechanical Turk to Studies Conducted Online and with Direct Recruitment

**DOI:** 10.1371/journal.pone.0121595

**Published:** 2015-04-14

**Authors:** Christoph Bartneck, Andreas Duenser, Elena Moltchanova, Karolina Zawieska

**Affiliations:** 1 University of Canterbury, Private Bag 4800, 8140 Christchurch, New Zealand; 2 CSIRO Computational Informatics Castray Esplanade, Hobart 7000, Tasmania, Australia; 3 Industrial Research Institute for Automation and Measurements (PIAP) Al. Jerozolimskie 202, 02-486 Warsaw, Poland; University of Vienna, AUSTRIA

## Abstract

Computer and internet based questionnaires have become a standard tool in Human-Computer Interaction research and other related fields, such as psychology and sociology. Amazon’s Mechanical Turk (AMT) service is a new method of recruiting participants and conducting certain types of experiments. This study compares whether participants recruited through AMT give different responses than participants recruited through an online forum or recruited directly on a university campus. Moreover, we compare whether a study conducted within AMT results in different responses compared to a study for which participants are recruited through AMT but which is conducted using an external online questionnaire service. The results of this study show that there is a statistical difference between results obtained from participants recruited through AMT compared to the results from the participant recruited on campus or through online forums. We do, however, argue that this difference is so small that it has no practical consequence. There was no significant difference between running the study within AMT compared to running it with an online questionnaire service. There was no significant difference between results obtained directly from within AMT compared to results obtained in the campus and online forum condition. This may suggest that AMT is a viable and economical option for recruiting participants and for conducting studies as setting up and running a study with AMT generally requires less effort and time compared to other frequently used methods. We discuss our findings as well as limitations of using AMT for empirical studies.

## Introduction

When conducting an experimental study, the way of recruiting participants for the study may already introduce a certain bias and hence great care must be taken in this process. Recruiting students for studies is very common in many disciplines (e.g. recruiting undergraduate psychology students for psychology experiments; recruiting computer science students for Human Computer Interaction (HCI) research) and potential bias due to this practice has been documented [1, 2]. Even when the scope of the recruitment is broadened to include participants from the entire campus, it cannot be assumed that results from such a study can be generalized to the general public [1, 3]. The main reasons for this so-called “convenience sampling” are not necessarily ignorance or ill will, but rather practical constraints. Recruiting a representative sample (e.g. general public, specific age groups, specific educational background, specific prior experiences) is difficult and hence expensive in terms of labour and necessary financial resources. Recruiting participants online has become a viable alternative to recruiting participants directly. Invitations to participate in E-mail and Web-based surveys can reach a large number of participants and considerably reduce recruitment costs and time [4, 5].

A convenient recruitment option is posting advertisements in mailing lists and online forums. A common practice is to address communication platforms in which the content of the questionnaire might be of relevance. If the questionnaire is, for example, about the prices of dairy products, then forums for farmers as well as forums for gourmets might be a source from which many responses can be elicited. But of course such a selection of communication platforms may bias the results by for example limiting the sample to a small self selected group (those who have online access, are interested in a specific topic, have signed up to and actively use the platform). The questionnaires can also conveniently be administered through the internet these days. The following section will provide an overview of online questionnaire research.

### Online questionnaires

According to Bulmer [6] a questionnaire is “any structured research instrument which is used to collect social research data in a face-to-face interview, self-completion survey, telephone interview or Web survey. It consists of a series of questions set out in a schedule, which may be on a form, on an interview schedule on paper, or on a Web page” [6] (also quoted in [7]). Over recent decades, survey techniques have gradually shifted from paper-and-pencil towards computerized surveys and online questionnaires. Computers were first used to assist telephone interviewing (CATI) and personal interviewing (CAPI) [8]. The rapid growth of the Internet has expanded research survey opportunities. By the early 1990s, researchers developed tools for conducting experiments over local computer networks [9] and eventually new web-based research techniques were introduced. The main motivation behind the usage of these new technologies for the recruitment and administration of questionnaires has been the increase of efficiency and effectiveness of the data gathering process. The main challenge has been, and still is, to ensure adequate data quality such as response rate, missing data or receiving truthful or honest responses.

Relationships between questionnaire mode, question content, question type and subjects’ responses are very complex [7]. However, several comparative studies have shown that there is no significant difference between the results obtained from computer-based surveys and those obtained from paper-and-pencil questionnaires [10, 11]. Some research findings demonstrate that online questionnaires improve data quality due to their lower missing responses rate and better internal consistency [10, 12]. It has also been noted that self-administered questionnaires increase participants’ willingness to disclose information about sensitive topics [7, 13] although no consensus has been reached on how different questionnaire modalities affect social desirability in responses [7]. Some authors argue that paper-based questionnaires generate a higher response rate [14, 15] and less extreme responses [16]. On the other hand the time for data collection is significantly shorter with online questionnaires than with traditional questionnaires [16]. Birnbaum and Mason [17, 18] discussed other advantages and disadvantages of web experiments, such as access to a diverse and otherwise inaccessible participant population, high statistical power due to access to large samples, cost savings of lab space and administration, and disadvantages like multiple submissions, self-selection, and drop out [19].

Researchers must consider different research constraints: self-administered Web-based surveys do not allow researchers to control the conditions under which surveys are completed [20]. Allowing people to complete a study at any time and in any environment potentially introduces confounding variables that might be unknown to the researcher [7]. This approach also makes it more difficult to answer questions that workers might have or clarify problems or misunderstandings.

### Amazon Mechanical Turk

Amazon’s Mechanical Turk (AMT) started in 2005 as an international crowdsourcing web service that enables task owners to distribute micro tasks to an anonymous workforce [21]. It can be used not only to recruit participants for a study, but also to administer the questionnaire itself. In essence, it is an online labour market where employees (workers) who meet predefined criteria are recruited by employers (requesters) for the execution of tasks (HITs—Human Intelligence Tasks) in exchange for a small wage (reward) [22].

Since it start, AMT has evolved and adapted to several changes in the US legislation. Currently, the requested need to be from the USA while the workers can be in many different countries (http://aws.amazon.com/mturk/faqs/). In 2012 when this study was originally conducted, this constraint was not yet in place and hence we were able to run the experiment while being in New Zealand. Some companies have emerged that offer the use of AMT for international requesters (http://mturkdata.com/).

The remuneration level depends on a number of HITs accomplished by AMT workers. According to one of the recent studies a median wage is equal to USD 1.38/hour [23]. AMT suggest an hourly rate of 6 USD, and many researchers are keen to pay their participants normal minimum wage in many developed countries. Although both requesters and workers are anonymous each of the AMT workers can be identified through the unique ID provided by Amazon [22].

Most of the Mechanical Turk workers are from the United States. However, the population of AMT workers has become more and more international with approximately 100,000 users from over 100 countries [24], including for example a considerable number from India (36% of AMT worker population by 2009). The demographic therefore is now wide spread and spans from workers who initially were mainly young, well-educated, and mostly female, with moderately high incomes to low-income workers in developing countries [25]. However, some of the studies report that the income level has shifted towards lower levels also among the U.S. workers on Mechanical Turk [22]. Due to the growing group of workers from India the number of male workers has been increasing while the average age has dropped from 32.9 in November 2008 to 31.6 in November 2009. The education levels remain high [25]. Berinsky et al. present more recent demographics of the AMT population and concluded that AMT workers are more representative of the general population and substantially less expensive to recruit [26].

There have been many questions raised about the representativeness of AMT samples and data quality. However, studies show that samples drawn from the population of U.S. workers (the main group of AMT workers) are comparable to samples from other subject pools, including standard Internet samples [26]. These samples also are much more diverse than the student population usually used in studies [27, 28], hence results may be often more generalizable than results from samples recruited through traditional methods [27]. In addition, AMT was found to be a reliable source of data and to diminish the potential for non-response error in online research [22, 27]. Some of the critics of AMT argue that due to the low financial reward, AMT workers pay little attention to the task they are accomplishing [29]. The average time to complete a task is higher in AMT studies compared to laboratory setting which indicates a possible problem of controlling the participants’ environment [5], although there are no set rules on how long a researcher should give subjects to complete tasks [30].

Nonetheless, it has been shown that AMT workers and participants who use traditional surveys do not differ in terms of attentiveness [31]. Besides, although most of the AMT workers participate in surveys because of financial incentives, many of them report that AMT also provides them entertainment in their free time [22]. Data quality seems to be independent from pay rates [27, 32] but AMT users tend to complete more tasks when they are paid more [5]. Other limitations to AMT studies include lack of possibility to exert control over participants’ environments compared to lab studies [27] and potentially lower English proficiency [31]. It is possible to ensure a higher level of English proficiency by adding a qualification test that needs to be passed before the worker is allowed to complete the actual task.

Examples of AMT being used for HCI related research include [5] who studied the feasibility for AMT for visual perception research. They did this by replicating earlier laboratory studies in AMT in which participants had to perform various visual judgements including spatial encoding and luminance contrast tasks. They could replicate their earlier results, indicating that AMT is a viable method for perception studies. They argue that the considerable cost and time savings that results from running a study in AMT could be reinvested in more participants and or conditions.

Kittur and colleagues [33] compared quantitative user ratings and qualitative feedback regarding the quality of Wikipedia articles given by highly experienced Wikipedia users to answers gathered through AMT. In the first experiment they found a medium to strong sized correlation (r = 0.50, p = 0.07) between AMT and expert answers. However, they also found a relatively high number of suspect or invalid responses (mainly received by a small minority of workers).

The second study was slightly changed and required workers to complete some questions that had verifiable answers before proceeding to rating the quality of the article. This resulted in a higher and significant correlation between AMT and expert ratings and considerably fewer invalid responses. Komarov et al. investigated if Graphical User Interfaces (GUIs) are evaluated differently by participants recruited thought AMT compared to participants in a laboratory setting [34]. Their results showed no significant difference.

AMT has one functional difference in comparison to many online questionnaire providers. AMT is designed to harvest the power of the parallel processing of tasks. Thousands of workers can work on the same batch of tasks in parallel. The requester defines how many different workers should execute a task, but the requester cannot require a worker to execute a certain number of tasks. This results in a situation in which AMT can guarantee that task A has been executed by x different workers, but these x workers might not be the same for task B. In traditional questionnaire tools it is assumed that one participant answers all the questions, meaning that the same participant that answered A also answered B. We refer to this type of conducting questionnaires within AMT as MT Batch Tasks.

One can work around this limitation of AMT by only posting a single task: a link to an online questionnaire. At the end of the questionnaire the worker receives a code that needs to be filled in as the answer for the single AMT task. This workaround does re-establish the traditional structure of the resulting data and processes. Given this high investment of time that participants make in completing a whole questionnaire, they might pay extra attention to the questionnaire. Since completing a full questionnaire is more work than completing a typical HIT, the participants would also be entitled to a higher payment. One could argue, however, that this workaround does not take advantage of the true power of AMT. In a previous study we set 722 tasks that each were to be executed by 30 different workers. This resulted in 21660 answers, which only took less than a day to obtain [35]. This demonstrates the enormous potential of AMT for conducting large scale studies with simple and repeatable tasks. But also the creation of questions is streamlined for large quantities in AMT. Instead of creating every question individually, as is common in most online questionnaire tools, AMT requires a template question and a list of stimuli. The questions are then created automatically by applying the template to the list of stimuli.

Given that one characteristic of AMT is the parallel processing of large sets of tasks we decided to include the administration of the questions directly within AMT. One of the disadvantages of MT batch tasks is that one can only ask one type of question per study. This means that it is not possible to ask the usual demographic questions such as age and gender and other mediator and moderator variables. It is also not possible to use conditional branching. These limitation is specific to the batch type of questionnaires in AMT. For other types of questionnaires, it is possible to ask different questions. In any case, the AMT population has been studied in detail in other research [25].

MT batch seems to be most appropriate for quickly gathering large amounts of data on relatively simple tasks involving stimulus-response type of tasks, personal judgments, categorizations of items and similar. Because it is difficult to control the specific population for recruitment, AMT is suitable for studies on the general population rather than specific sub-groups. This either has to be considered when considering using AMT, designing tasks with AMT or at least when analyzing and interpreting the acquired data. This also means that study setups that involve more elaborate designs, several tasks to complete, monitoring participants, or controlling certain moderator variables would be difficult or hardly possible to carry out with MT batch. Some of these limitations though can be overcome by using AMT for recruitment only.

Because, arguably, MT participants might be tempted to cheat, we have visually checked responses for obvious patterns (such as repeatedly pressing the same button). We have also checked the individual response sequences for randomness by calculating pairwise agreement as the proportion of minifigures, which the two individuals have rated identically. As mentioned above, in the MT sample this proportion varied from 20.21% to 86.17% with the average of 56.40%. Note, that in the assisted sample, where we are sure of the absence of bots, the respective range was 25.53%-80.85%, with the average of 50.56%. According to basic probability theory, the expected percentage of agreement with a random sequence of length 94 is 1/6 with standard error of $\sqrt{\frac{\frac{1}{6}*(1-\frac{1}{6})}{94}}$. None of our evaluated pairwise agreement measures fell into the interval, implying that no responses were given at random.

### Research Question

Several studies have been carried out in order to compare the results obtained through AMT with results obtained through other methods, such as pencil-and-paper, computerized and online questionnaires. Although some indications are available showing that data from AMT studies is consistent with data from those conducted in a physical lab [9, 18, 31], the use of AMT for research purposes remains subject of academic discussion. One of the open questions is for what types of tasks AMT might be a suitable solution. Secondly, it is unclear if conducting studies within AMT through a batch tasks results in different responses.

This study examines how different methods of recruiting may influence the similarity of the results obtained. For this we compare how participants rate the emotional expressions on LEGO Minifigure faces. The three recruitment conditions in this study are AMT, university campus and online forums. In addition, we investigated whether conducting a study within AMT results in different responses compared to a study were AMT is only used for the recruitment but the questionnaire itself is hosted at an external online questionnaire service. We also present and discuss our findings on response rates, time required to collect the data and other practical advantages and disadvantages of the different methods.

Another question we address in this study is the reliability of results obtained through AMT. A questionnaire administered at a certain point in time might results in different results when administered at a later point in time due to the fact that other people might have answered. It is unclear if the great variety of participants active in AMT might introduce a bias.

## Method

We employed a between-subject design in which the recruitment method was the independent factor. The three conditions were AMT, campus and online. Moreover we ran another between-subject trial in which the questionnaire platform, MT or MT Batch Tasks, was the independent factor. In the MT Batch condition, the questions were administered within AMT and in the MT condition the questions were administered through an external questionnaire service. To test the replicability of AMT approach we sampled the MT and the MT Batch conditions twice. The consent procedure has been managed through the Amazon Mechanical Turk Participation Agreement. Running a study through Amazon Mechanical Turk is slightly different from common psychological studies. As an experimenter, you have no direct contact to the participants and the whole study has to comply to the Amazon Mechanical Turk Participation Agreement. Our study did comply to this agreement and was therefore allowed to run through their system. This study was considered to be of extreme low risk by the lab and hence was exempt from the ethics approval procedure of the university, which includes the need to inquire formal consent forms. The AMT participants remain anonymous automatically due to the setup of AMT. We did not ask for any data that would identify participants. The same holds true for the participants that were recruited online and on the campus.

### Previous study

We recently performed an analysis of 722 Minifigure faces and were able to show that they offer a wide variety of emotional expressions [35]. This study uses a subset of the data obtained in the mentioned study for the MT condition presented here. We randomly selected 100 Minifigures, but six of them did not have a face and were therefore removed from the sample. The remaining 94 Minifigures were used to test participant responses in this study.

### Design

**Fig 1:**
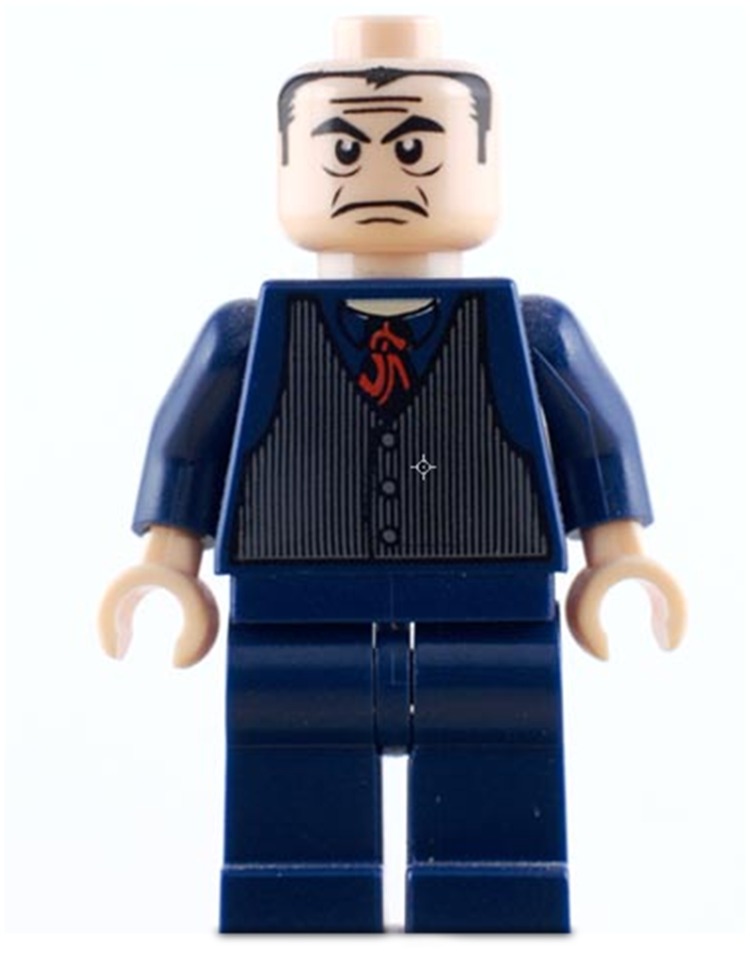
A LEGO Minifigure.

Since participants in the online condition may have different cultural backgrounds, we had to develop a questionnaire that was robust against cultural differences. At the same time, we could not assume that all participants would be native English speakers. The questionnaire therefore needed to use as little written text as possible. Based on these constraints, we selected the recognition of emotions in faces as a suitable task for the questionnaire. Ekman [36] demonstrated that the perception of emotions (happiness, anger, sadness, disgust, surprise and fear) is universal, meaning that the perception is constant across cultures. However, there might still be a preference for faces from the same ethnic group [37]. We therefore decided to use more abstract faces. The LEGO company has been producing little figures, called Minifigures, since the early 1970s and these are available in more than 130 countries (see Fig 1). There are of course many other application areas for which AMT could be used, but clearly it is not possible to consider every application scenario in just one study. We hope that more research will take the study at hand as an inspiration to validate AMT in different contexts.

LEGO products and figurines are very common in many countries and there is a good chance that many people around the globe may be familiar with the faces of LEGO Minifigures.

#### Questionnaires

**Fig 2:**
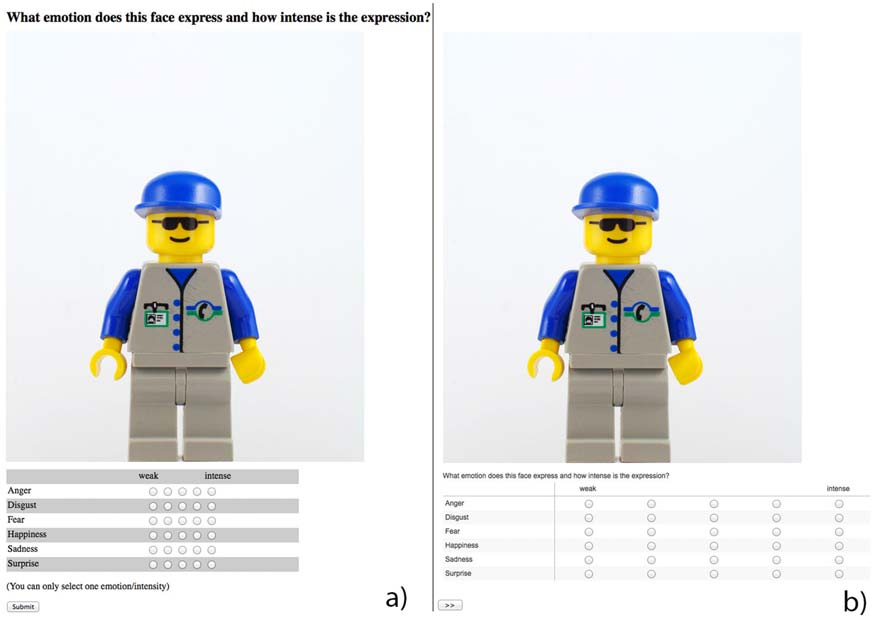
Questionnaire layout a) MT b) Qualtrics.

The questionnaire asked participants to rate the emotional expression of 94 different LEGO Minifigure faces. For the three recruitment conditions (MT, Campus, Online), we used the Qualtrics service. For the MT Batch condition the questionnaire was presented within the AMT system while the MT condition used again the Qualtrics service. Both administration tools used the exact same faces, the same functionality and the same layout (see Fig 2).

#### Process

The task for the participants in all conditions was to rate the exact same set of 94 LEGO Minifigures. The Minifigures were randomly selected from a larger set of 722 Minifigures used in a previous study [35]. Each Minifigure had to be rated by the participants to represent one of six emotions (Anger, Disgust, Fear, Sadness, Happiness, Surprise) with one out of five intensities (weak (1) to intense (5)). The questionnaire only allowed for one combination of emotional category and intensity. If, for example, the participant first considers a face to express fear at intensity level 4 and then changes his/her mind to surprise at intensity level 3, then only the later would be recorded.

For the campus condition, we set up a laptop computer in the central library (University of Canterbury, New Zealand) and asked students to participate in our study. Participants were approached in person on the campus and information about the study was posted on one of the University’s social network sites. Unlike in the MT study, no financial reward was offered to participants in the campus or online conditions. While it would have been easy to reward participants in the campus condition, it was not possible for us to offer a financial reward to the online participants due to University constraints processing micro payments via the internet. We do not believe that this would strongly bias the results since it has been shown that the data quality is independent from the pay rates [27, 32] and that financial incentives do not constitute the only motivation for participating in experiments but also entertainment, leisure or finding meaningful ways of spending free time [22].

We estimate that it took approximately 30 minutes to complete the questionnaire. First, the participants read an instruction sheet before they could ask any questions. Next, the experimenter started the questionnaire on the computer administered using the Qualtrics service.

For the online condition, we advertised the questionnaire in popular LEGO forums, such as Brickset.com and Eurobricks.com. The participants started with the instructions displayed on a web page before entering the questionnaire on the Qualtrics server. The questionnaire was exactly the same as for the campus condition. We have chosen these forums for practicality reasons with the aim of getting a big enough sample of participants, with the expectation that there is a high chance that these forum members participate in such a study. While this recruitment approach might introduce some bias such as self selection bias (due to the participant’s interest in LEGO) we share a potential lack of generalizability with other recruitment approaches frequently found in psychological research. In these, convenience samples are often taken from a University student population (e.g. psychology students).

In the MT condition, the participants first read the instructions. They then rated as many Minifigures as they wanted, with the limitations that they could not rate the same Minifigures twice and that each Minifigure got rated by at least 30 participants and at most 35. Since there is no ground truth for this task, we were not able to reject any responses from HIT workers. We did check for regular patterns that would have revealed fraudulent behavior.

For the MT condition we used AMT only as a recruitment tool. The task for the workers was to click on a link to the Qualtrics questionnaire. After the workers filled in the questionnaire they received a confirmation code that they had to copy back into the MT task as a proof for the completion of the questionnaire. For both conditions, the MT and the MT Batch, we required participants to be located in the USA, which ensured that they would be very likely to understand English.

Most people have considerable experience in reading the emotional expression of human faces, even abstracted ones. We are generally able to understand the expressions of comic characters as well [38]. Given the millions of LEGO Minifigures sold, we may also assume a certain familiarity with LEGO faces. We therefore assumed that the size of our samples was sufficiently large to be resilient against possible biases. It is unlikely that we accidentally sampled a large number of participants that might have a psychological disorder that might inhibit their skill of judging faces. We did not filter participants based on their prior experience with LEGO since we assumed that the task was sufficiently easy for everybody to complete.

Table 1 summarizes several aspects of the four recruitment and survey conditions used in this study. The conditions represent typical study scenarios used in HCI research. The methods differ in several aspects such as location, duration, approaches and systems being used for recruiting participants and for collecting the data, sample size and type, as well as reward provided for participation. With some of these approaches, certain aspects remain difficult to assess, for example when running a study within MT Batch it is difficult to get clear demographic data from the participants as well as the location in which the participants work on the given tasks. While demographics are easier to assess with the other methods, study location is also difficult to control with online studies. On the other hand, it is much easier with online and AMT to easily recruit large sample sizes and collect data in a short amount of time. The anonymity provided by online and AMTs can also be an advantage when sensitive data is assessed, and can increase the likelihood of respondents giving more honest or less socially desirable responses [14, 39]. The fact that the methods vary in more than one aspect means that we do not just investigate one single dimension by comparing the three methods. We put our research into the actual context of use, thereby providing insights with higher ecological validity.

**Table 1:** Comparison of the conditions on various aspects as listed in the leftmost column.

	MT	MT Batch	Campus	Online
Country	USA	USA	New Zealand	Unknown
Location	Online	Online	Univ. Library	Online
Duration	∼ 30 minutes	variable	∼ 30 minutes	∼ 30 minutes
System	Qualtrics	AMT	Qualtrics	Qualtrics
Task Type	Questionnaire	Questionnaire	Questionnaire	Questionnaire
Recruitment	AMT	AMT	in person	forums
Participants	variable (see [22])	variable (see [22])	Univ. students	Unknown
Sample size	29 and 31	209 and 83	32	185
Reward	Money	Money	None	None

The MT and MT Batch conditions were run twice each. The first sample was used for comparisons between various conditions, and the second sample was used to test for replicability of the results.

### Participants

The ratings given by a total of 569 participants were recorded. Of those, 209 and 83 were obtained from the two runs of the MT Batch procedure. 29 and 31 participants were recruited for the two runs of the MT condition, 32 for the campus, and 185 for the online condition. Since the second samples of MT and MT Batch were used for validation only, a total of 455 were used in the initial comparison. The participation statistics for each procedure is shown in the Table 2. A total of 17545 individual assessments were thus obtained. Notice that the campus and MT condition have a similar response rate. But only in the MT Batch condition it can be guaranteed that each stimulus is rated exactly 30 times.

### Statistical Methods

The participants had to categorize each of the Minifigures into one of the six emotional categories. There is no correct answer or ground truth to which the participants’ scores can be compared. Therefore we cannot say that one method is better than another, we can only say whether the methods give similar results or not.

In this analysis we have chosen to focus on the emotion classification data (i.e., Anger, Disgust, Fear, Happiness, Sadness, Surprise) omitting the intensity. The reason for this is that the two variables—emotion category and intensity—form a single bivariate response and should ideally be analysed as such, since the two are not independent. But although various methods exist for analysis of simple contingency tables [40], no framework has been developed as yet to also take into account other covariates as well as random effects, which is necessary as described below.

Categorical data is commonly modelled using multinomial regression with a standard logistic link model. The additional fact that the same person was allowed to assess multiple Minifigures can be taken into account by introducing both fixed and random effects. We therefore fitted a mixed effects multinomial logistic regression model [41]. Due to the large number of parameters (94Minifigures ×6categories ×3methods +455 participants) and different number of responses per person we chose to use the Bayesian framework for our analysis [40], which is more flexible and relatively easy tp implement in the freely available WinBUGS software [42].

In classical statistics, the data are assumed to result from an experiment infinitely repeatable under unchanging conditions. The observed frequencies are used to estimate the unknown parameters; hence the alternative name “*frequentist*” statistics. In the Bayesian framework, a datum is supposed to result from a unique (i.e. non-repeatable), experiment, and the unknown parameter is viewed as a realization from an unobserved distribution, which is the object of the inference. Bayesian inference is very flexible with regard to model structure and assumptions. This allows for the seamless inclusion of prior beliefs, if there are any, and for efficient updating of the results once the new experiments are conducted. When no specific information is available on parameters beforehand, and the prior distributions are thus non-informative, a Bayesian approach will usually produce results similar if not identical to those produced by the classical approach [43].

The estimation usually requires the use of Markov Chain Monte Carlo (MCMC) numerical methods. Bayesian inference is not without controversy, mostly pertaining to the choice of priors and the assessment of MCMC convergence. However, during the last decades it has become a widely accepted methodology, especially when dealing with unconventional complex multiparameter models.

Mathematically, our mixed effects multinomial logistic model can be described as follows.

Let *Y*_*mfpk*_ = 1 if the person *p* assessing Minifigure *f* through process *m* has assigned emotion *k* to it, and *Y*_*mfpk*_ = 0 otherwise. Here, *m* = 1,2,3, *f* = 1,…, 94, and *p* = 1,…455, and, naturally, not all combinations are recorded, giving in effect a so-called ragged array of observations. Then, for each observed combination *mfp*, we have

$$\begin{array}{c} Y_{mfp•}∼Multinomial\left(1,\pi_{mfp•}\right) \end{array}$$

where *π*_*mfp*•_ is the vector of probabilities of assigning each of the 6 emotions to the Minifigure *f* by person *p* through method *m*. The traditional link function can then be modeled as

$$\begin{array}{c} \pi_{mfpk}=\frac{exp(\eta_{mfpk})}{\sum_{k}exp(\eta_{mfpk})} \end{array}$$

where, for the purposes of model identifiability

$$\begin{array}{c} \eta_{mfp1}=0\;\forall m,f,p \end{array}$$

and otherwise

$$\begin{array}{c} \eta_{mfpk}=\alpha_{mfk}+\epsilon_{mfpk} \end{array}$$

with

$$\begin{array}{c} \epsilon_{mfpk}∼N\left(0,\tau_{m}\right) \end{array}$$

and

$$\begin{array}{c} \alpha_{mfk}∼N\left(0,\omega \right) \end{array}$$

Here, *α* are the fixed effects of method and Minifigure, whereas *ε* are random effects of personal choice.

We have also found that it is usually possible to simplify the above regression structure to

$$\begin{array}{c} \eta_{mfpk}=\alpha_{mfk}+\epsilon_{pk} \end{array}$$

with

$$\begin{array}{c} \epsilon_{pk}∼N\left(0,\tau \right)\cdot \end{array}$$

**Fig 3:**
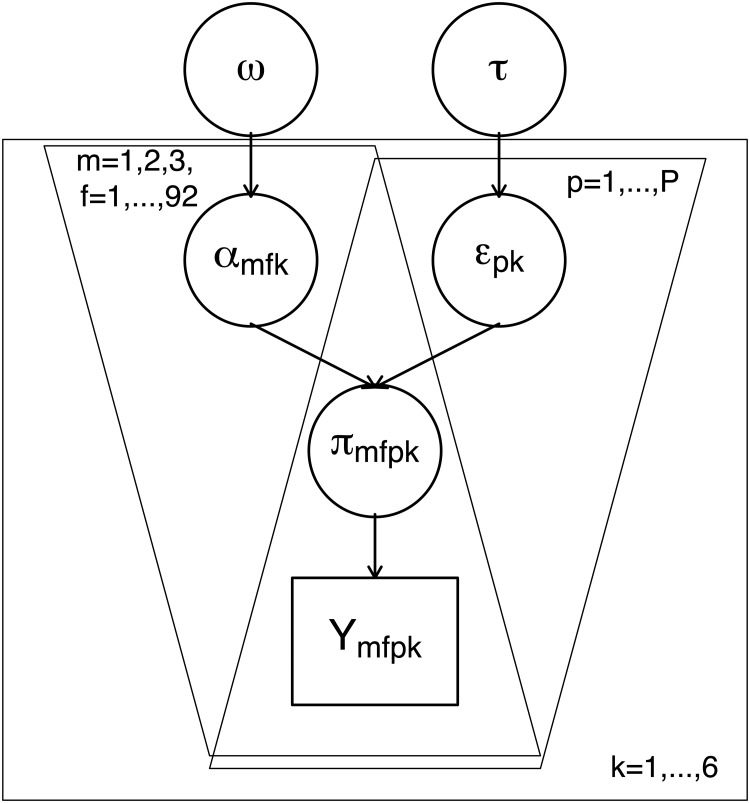
Plate Diagram for the simplified Bayesian multinomial logistic random effects model.

The above model, illustrated in Fig 3, has been set up within a Bayesian framework. The fixed effects *α* were assigned non-informative Gaussian priors, whereas the precision (inverse variance) parameters *τ*_*m*_ were given vague Gamma priors. The model was estimated using WinBUGS software [42]. 10000 iterations with a 5000 burn-in were run. The convergence was assessed visually.

Minifigure-specific method comparisons were also made by fitting the model with a common probability vector and the model with method-specific probability vectors to the aggregated data. A standard non-informative Dirichlet prior with parameter *a* = [111111] has been used.

The Bayesian Deviance Information Criterion (DIC) [44] was used for model comparison. Similar to the classical Akaike Information Criterion (AIC), it can be calculated for any parametric model to provide a measure of balance between parsimony and fit. Of two models, the one with the smaller DIC is considered the better one. The degree of superiority of one model over the other may thus be reported in terms of the difference between the two DICs. The general guidelines are that DIC difference of 5–10 may be considered substantial, while those above 10 definitely rule out the model with higher DIC (http://www.mrc-bsu.cam.ac.uk/software/bugs/the-bugs-project-dic/#q9). In this study, the null model always referes to the one where no distinction was made between the methods (i.e., $\eta_{mfpk}={\tilde{\eta }}_{fpk}$ for all *m*), and the alternative model was the more statistically complext one with method-specific parameters. We denote the associated difference in DICs: Δ*DIC*. A positive Δ*DIC* means that the more complex model is better, i.e., supports distinction between the methods.

Note, that an even simpler version of the link function may be considered:

$$\begin{array}{c} \eta_{mfpk}=\beta_{mk}+\gamma_{fk}+\epsilon_{pk} \end{array}$$

where the potential effect of method on the emotion assessment is assumed to be the same for all the Minifigures. This would considerably decrease the number of parameters, but we have found that the more complex version is statistically justified (Δ*DIC* = 409.1).

We have used the same method to investigate replicability of the MT and the MT Batch by comparing the two samples available for each condition. In this case, the subscript *m* = 1,2 refers to the first and the second samples respectively.

## Results

We conducted three analyses to answer the main research questions. The data on which the analyses is based on is available (http://bartneck.de/projects/research/emotionFace/archive_data.zip). Before going into the details of answering the research questions, we first want to show some more general results.

### Distribution of Emotions

**Fig 4:**
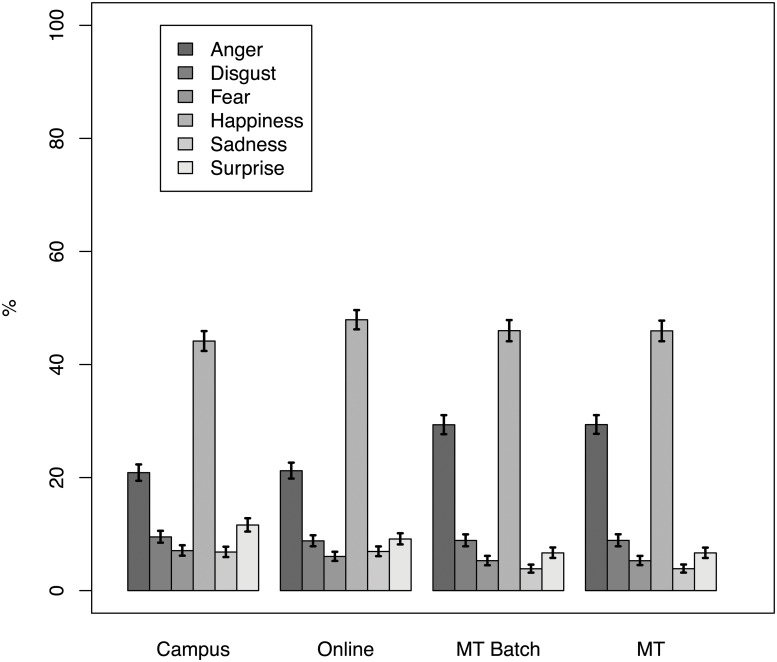
Estimated average distribution of emotional assignment by method (campus, online, MT, MT Batch).

The distribution of emotions was not uniform. Fig 4 shows that happiness and anger are the most frequent emotions expressed.

### Response Rates

The completion rate in the different recruitment conditions shows clear differences. The participants in the campus condition were asked in person to participate and the experimenter was available throughout the study. This resulted in mostly completely filled-in questionnaires (see Table 2). In the online condition, the participants seemed less committed to complete the survey. On average, the online participants completed only one third of the questionnaire. However, with only a few posts in online forums, we were able to elicit five times more participants compared to the campus condition. In the MT Batch condition, we also got fewer complete datasets for the 94 Minifigures per participant. The incomplete datasets in the MT Batch condition were mainly due to the way data was collected through AMT and because we only took a subset of the complete dataset. However, it was possible to acquire a similar number of total responses as in the campus condition in only a fraction of the time. It took only a few hours to recruit all participants for the MT and MT Batch condition, while it took several days to recruit all the participants for the online and campus condition.

**Table 2:** Number of responses by condition.

	min	max	median	mean
MT	94	94	94	94
MT Batch	1	94	6	14.5
campus	92	94	94	93.8
online	1	94	20	36.5

### Recruitment

When comparing the three recruitment conditions we found strong evidence for MT being different from the other two (Δ*DIC* = 318.9). The online and campus condition did not signficantly differ (Δ*DIC* = −241.3).

We conducted a pairwise comparison across all three recruitment conditions to see how many Minifigures were rated differently. In each case, we have run a model where the parameters were method specific and a model where the parameters were global and used DIC to see whether the first model was better, i.e. whether there was a difference between the methods. The summary of pairwise comparisons is shown in Table 3. It demonstrates that while the results of campus and online processes were generally in agreement (differences for 4% of the Minifigures), the results of the MT condition were only significantly different for around 15–20% of the Minifigures. Most of the figures were predominantly classified as either happy (60%) or angry (27%).

**Table 3:** The numbers of Minifigures, for which the three methods produce strongly contradictory results (Δ*DIC* ≥ 10), mildly contradictory results (5 < Δ*DIC* ≤ 10) and agree (Δ*DIC* ≤ 5). The rows where two methods are listed test for the respective pairwise difference. The rows where three methods are listed test for at least one being different from the rest.

	Strongly Contradictory	Mildly Contradictory	Agree
	Δ*DIC* ≥ 10	5 < Δ*DIC* ≤ 10	Δ*DIC* ≤ 5
MT Batch vs. Campus	14	18	62
MT Batch vs. Online	19	19	56
Campus vs Online	4	18	72
MT Batch vs. Online vs. Campus	39	16	39
MT vs. Campus	33	19	42
MT vs. Online	28	25	41
MT vs. Campus vs. Online	39	13	42
MT vs. MT Batch	11	5	78

### Comparison of MT Batch to Online and Campus

Despite the fact that the MT Batch condition varies in more than one way from the online and campus condition (see Table 1), we were still interested in comparing them. When fitting the overall mixed effects logistic regression model, however, the null model (no effect of method) was significantly better than the one with the method effect (Δ*DIC* = −214.7). This means that there was no significant difference between MT Batch, online and campus conditions. Fig 4 shows how little the three conditions differ.

### Response Consistency

In order to analyse whether there is an assessment order or learning effect, we have analysed whether the way Minifigures are assessed by a participant changes over time. Therefore we have looked at figurine-specific total and generalised sample variance vs. the order of the figurine presentation for online, campus and MT, but we found no trends. Neither did we find trends in DIC-differences, which would indicate consistent increase or decrease of agreement either within or between the methods. Therefore there is no evidence of either learning effect or loss of concentration effect.

We have calculated two indices in order to get a better understanding of the variability of answers across the conditions (Campus, Online, MT Batch, MT): (a) how many emotions are ascribed to a Minifigure on average within each group and (b) the highest agreement percentage, i.e. percentage of raters for the most commonly rated emotion per Minifigure. Results showed that on average the MT group agreed more often and with a higher percentage (3.6 emotions assigned on average to each Minifigure, 70% on average agreed on the most frequent emotion) than the other groups. The corresponding figures for MT Batch are 3.9 emotions per Minifigure and 68% agreement. The campus and online conditions both resulted in 4.0 emotions on averaged assigned to each figurine with the agreement percentages of 64% and 67% respectively. This indicates that ratings from participants in both MT groups were somewhat more homogeneous than ratings in the other two groups.

**Fig 5:**
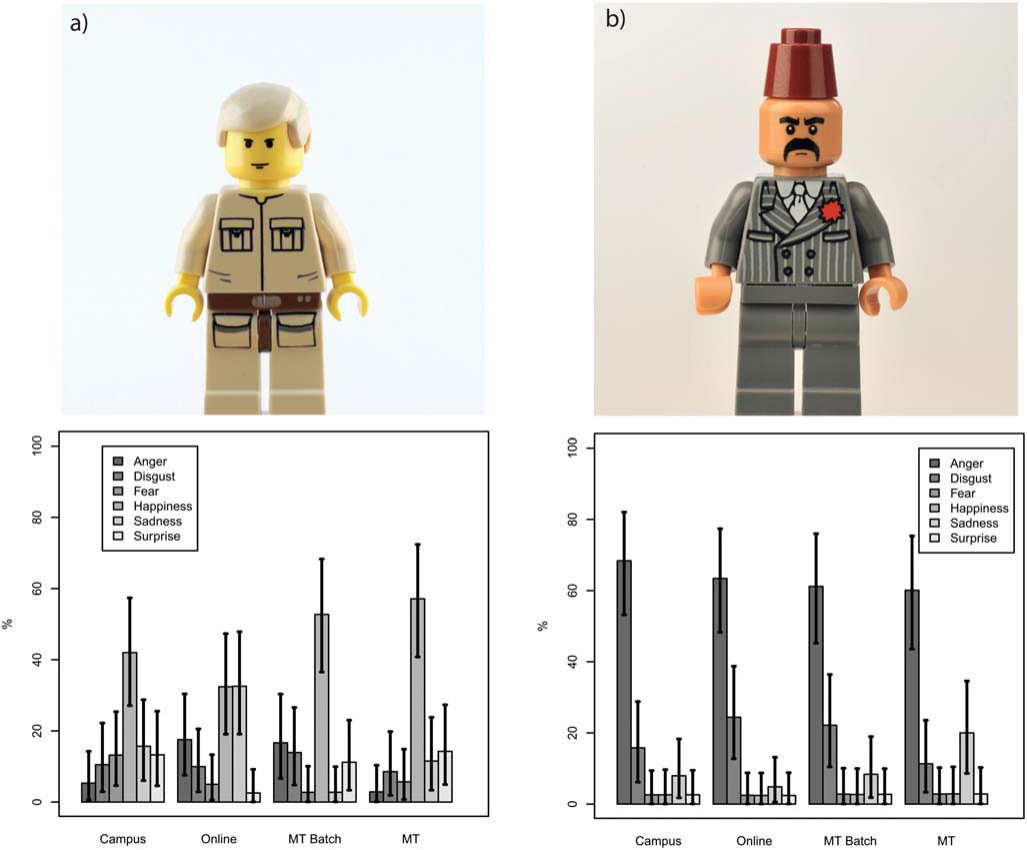
Frequencies of emotional categories for two Minifigures a) Minifigure 03753 b) Minifigure 00948

The variability of assessment can be demonstrated by looking at the ratings for two example Minifigures (see Fig 5). The Minifigure with the ID 00948 is an example of a Minifigure for which the resulting distributions of emotion assignment are similar for the three recruitment conditions. The Minifigure with the ID 03753 is an example for which the assignments across the three recruitment conditions are very different. We speculate that the different evaluations of ID 03753 are based on the ambiguity of the expression. The eyebrows may or may not express anger and it is also not clear if there is a smile present or not.

The person-specific effects were found to be highly significant. We compared the models with and without the person-specific effects and found the latter to be markedly worse (Δ*DIC* = 1219). This means that the mixed-effects model rather than a simpler fixed-effects only model should certainly be applied despite the greater implementation effort and the longer computing time.

### Replicability and Simililarity of MT Conditions

Two samples of both, the MT and the MT Batch conditions were selected. We tested the replicability of each by comparing a model that distinguished the samples to a model treating them as one pooled sample. The latter was better for both the MT (Δ*DIC* = 332.0) and the MT Batch (Δ*DIC* = 269.39).

## Discussion

The results of our analysis show that there is a significant difference of results obtained when recruiting participants through Mechanical Turk (MT conditions) in comparison to when participants were recruited on campus or through online forums. This result is not inline with previous work that showed that the results obtained from AMT are similar to other recruitment methods [5, 34]. There was no significant difference between the online and campus recruitment conditions.

Although we did find a statisiticaly difference between MT and the online and campus recruitment methods, we still need to consider whether this difference has any practical implications. Unfortunately, there is no easily interpretable statistic to summarize a difference between two multinominal distributions. However, Fig 4 shows that overall the four observed frequency distributions look very similar.

One population characteristic that potentially could have some influence is that participants in the MT sample most likely come from a wider, more dispersed population [25]. The Campus sample was drawn from a smaller population (University students) which share more common characteristics. The online sample was recruited through popular LEGO forums. Although we did not collect specific demographic data, we speculate that this sample might be from a somewhat more varied population than the Campus sample. One factor that these participants share though is that they are most likely more used to and interested in LEGO Minifigures than others. A less homogeneous population, not sharing as many common characteristics or interests such as MTs could potentially produce answers with higher variability. Interestingly we found evidence that suggests the opposite to be the case. Our results indicate that ratings obtained by the MT group are more homogeneous than the rating from the other groups. MT participants on average showed less variation in assigning different emotions to particular Minifigures and showed higher agreement for the most commonly rated emotion per Minifigure. That means that the MT group on average agreed more on which emotion to assign to a Minifigure.

Another explanation for the slight differences in assessment between the groups could be found in participants’ commitment to complete the tasks. AMT allows an experimenter to quickly obtain results from a large number of participants. However, with all kinds of computer-assisted surveying there might be a risk of losing control over the quality of the responses [39]. Generally the less control through or presence of an experimenter during task completion, the less pressure seems to be present on the participant to complete the task. The lack of an experimenter being present on the other hand can make the participants less likely to give socially desirable responses and because of the provided anonymity, people could feel safer to give honest answers [14, 39].

There was no significant difference between the results obtained through AMT directly (MT Batch condition) and results obtained when AMT was only used to recruit participants (MT condition). We did show that MT is statistically different from Campus and Online and that MT is not statistically different from MT Batch. This does not, however, mean that MT Batch is also statisitically different from Campus and Online. When we compare the results from the online and campus conditions to the MT Batch condition then we find no significant difference. The null model was significantly better than the one with the assumption that there would be a difference (Δ*DIC* = −214.7). Data obtained through MT Batch are not fundamentally different from the other two methods.

This is not necessarily counterintuitive in view of the fact that we have found random person-specific effects to have significant effect on the model (Δ = 1219) and the MT sample provides more repeated observations per person than an MT Batch sample allowing to better estimate the uncertainty and providing more power to detect differences. The observation is similar to fitting a fixed-effects-only and a mixed effects model in the classical framework.

The results of this study lead us to believe that MT Batch is a viable option for recruiting and conducting studies that do not need strict control over the study population (e.g. studies with general population as target), or studies that do not require that all tasks are completed by the same participants such as in our study. Complete sets of data can be obtained directly in MT Batch by including responses from multiple participants. This study adds to the general validity assessment for studies conducted in AMT. Previous studies already have evaluated AMT as a pure recruitment tool, but our study provides insights into conducting studies within the AMT environment.

Not surprisingly, the data is also not identical. MT Batch is different from campus for 15% of the Minifigure assessments and different from online for 20% of Minifigure ratings. Campus is different from online in only 4% of cases. This suggest that, on a Minifigure-by-Minifigure basis, results gathered from groups recruited directly by an experimenter and from groups recruited online are similar more often than results gathered from MT Batch participants. In some specific cases the assessment method can produce very different results such as illustrated in Fig 5. Thus while the recruitment method does not matter overall, MT Batch can sometimes produce slightly different results.

We have to keep in mind that the Minifigure-by-Minifigure comparison does not take inter-personal differences into account. Certain participants might have rated Minifigures in general more positively than others or might have answered with some bias towards certain emotions (e.g. often rating Minifigures as being angry) or emotional strength (e.g. not giving extreme judgments). Although the emotion perception is culture-independent, judging emotions is nevertheless a subjective task and inter-personal differences are to be expected to some degree.

The unbalanced study design might have contributed to the potential dissimilarity between the results of the individual comparisons and those of the global mixed-effects model. While some participants have rated 94 figurines, providing us with a good idea of their general marking tendencies, others (especially the MT Batch respondents) have rated only a few. Our analysis showed that there was no learning effect. Participants did not change the responses based on the fact that they have already rated a certain number of faces. This means that the unbalanced design is unlikely to have affected the results.

We may also speculate about other reasons that might have contributed to the observed differences in the Minifigure by Minifigure comparison. While we cannot rule out the possibility that some of the differences in emotion assessment between the groups could be due to differences in sample populations, the three populations in this study can be assumed to be not fundamentally different. Furthermore as mentioned earlier, emotion perception, especially for abstract faces such as the ones used in this study, is culturally independent.

We could not find evidence for participants from either condition to be more likely to adopt certain answering strategies such as pattern answering, tendencies towards central or extreme answering options or randomly clicking on answering options.

We have visually checked responses for obvious patterns (such as repeatedly pressing the same button). We have also checked the individual response sequences for randomness by calculating pairwise agreement as the proportion of minifigures, which the two individuals have rated identically. In the MT sample this proportion varied from 20.21% to 86.17% with the average of 56.40%. Note, that in the assisted sample, where we are sure of the absence of bots, the respective range was 25.53%-80.85%, with the average of 50.56%. According to basic probability theory, the expected percentage of agreement with a random sequence of length 94 is 1/6 with standard error of $\sqrt{(}\frac{\frac{1}{6}*(1-\frac{1}{6})}{94}$. None of our evaluated pairwise agreement measures fell into the interval, implying that no responses were given at random. But we found that commitment to complete the survey varies between conditions. In the campus condition participants usually finished the survey with few missing data points. In the online condition the dropout rate was higher and for MT Batch we got even more incomplete datasets. However, because of the way data is collected through MT Batch, incomplete datasets could have been the result of other factors rather than commitment. When conducting a study in AMT the experimenters cannot include restrictions that ensure that participants have to complete all tasks. The MT data used in this study is a subset of the data used in a previous study that comprised a total of 722 Minifigures [35]. Participants could rate as many Minifigures as they pleased. We included an automatic function to ensure that each task would get completed by 30 different participants. Thus, some participants might not have rated one of the 94 Minifigures relevant for the present comparison because they either chose not to complete all tasks, or because one of the Minifigures already had all required ratings.

With a high amount of Minifigures being rated, there is a chance for bias due to learning or loss of concentration. This could potentially affect the comparison results because the participants in the different conditions did not all rate the same amount of Minifigures. However, we have found no trend for either increase or decrease of agreement within or between the methods. This indicates that participants do not obviously change their ratings over time.

Another consideration we would like to share is the observed recruitment effort. Recruiting participants and conducting the study is most labour intensive for the campus condition. One researcher had to spend several days in the library to solicit a sufficient number of participants. The recruitment and data collection effort necessary for the online and two MT conditions is considerably less and a sufficient number of responses can be acquired in a very short time. We gathered the responses in the two MT conditions in a few hours, without the need for an experimenter being present. Although in MT Batch operation one participant might not complete all tasks, AMT does allow the experimenter to specify the exact number of responses per task, in our case 30 ratings per Minifigure. AMT can also be used as just a recruitment tool as we have done for the MT condition.

The possibly greatest advantage of running a batch questionnaire in AMT is that it is very easy to setup a large number of items to be rated. After defining a template for the question the user only has to provide a table with the stimuli, such as a list or URL for the images to be rated. AMT then automatically creates all the questions for the batch and ensures that every item is rated for at least as often as specified. Moreover, AMT even offers an Application Programming Interface (API) that enables researches to completely automate the creation of batches. This makes MT Batch a powerful tool for large sets of items that need to be rated.

### Limitations

Overall we have found Mechanical Turk to be a valuable tool for gathering experimental data. The MT Batch method produces results comparable to other commonly used data collection methods. However, there are certain limitations.

The number of participants in each condition was between 29 and 32. This was due to the desire to keep the group sizes equal. A limiting factor for the sample size is the effort involved with data collection and the number of volunteers available on-campus. Still these numbers were large enough to find statistical differences between the groups. Further studies with larger groups would be needed to explore the differences in more details as well as to assess the representativeness of the samples.

In this study we have only been able to test one type of questionnaire. Therefore it is not clear at this stage whether our findings generalise to other tasks. It is conceivable that not every task might be suitable for MT Batch. However, it is likely that more text based questionnaires can also be used in MT Batch if a qualifying test is used to filter out unsuitable participants. But it is always possible to fall back on using AMT only as a recruitment tool and not as a tool to execute the questionnaire itself. Such an approach allows the experimenter to filter participants even prior to administering the core questionnaire.

For tasks that involve gathering objective data, such as response times and error rates, AMT might be a very suitable tool, in particular if the goal is to test the general population. The lack of control in recruitment and data collection procedure with AMT means that the experimenter has less influence on strictly controlling the study parameters and necessary variables. This potentially introduces more random noise or unwanted effects from uncontrolled variables. Therefore experimental results always have to be interpreted rather carefully.

When conducting the study within AMT as a batch task, it cannot be guaranteed that all participants complete all the tasks. Studies that want to monitor or control a learning effect, for example, would not be suitable for an MT batch approach. For these types of studies, AMT can best be used as a recruitment tool.

With AMT it is difficult to gather demographic data. While demographics can be gathered when using AMT only as recruitment tool, questions regarding the participant’s background, gender, age etc. cannot be asked when running studies in MT batch mode. The AMT population has been studied at large in other research. Berinsky et al [26] for example showed that samples from AMT are “often more representative of the U.S. population than in-person convenience samples“. Still, researchers cannot clearly determine the specific composition of the actual sample in any given MT bath based study. This might be problematic for studies that depend on for example culture or gender dependent issues. If such factors are of importance AMT still could be used as a recruitment tool, allowing to collect demographic data which can be used for further analysis of screening and filtering of participants. Furthermore, AMT workers come from different countries around the globe. The financial reward offered therefore presumably could be a big or rather minimal incentive depending on the average wage in the AMT workers home country. Previous research has shown that financial reward is not the only motivation to participate in studies, but there still could be considerable differences in people’s motivation for participation. One means of controlling for this is filtering AMT workers based on their location.

Another limitation is that only one task can be set up for the AMT worker to complete. In our case this was a presentation of a set of stimuli and a set of response options. While the stimuli can be changed, the type of task cannot be altered. This obviously is suitable for stimulus-response type experiments or studies that do not require a more complex set of questions or stimulus-response sets. But this could be a limiting factor for researchers who are interested in covering a wider set of factors or questions. This includes different types of tasks or items that might have a potential confounding effect. Researchers hence should be aware of this and design AMT studies appropriately.

While it is very easy with AMT to set up a large number of items and gather data from many participants in a very short time frame, it can be more difficult to get complete data sets unless the experimental setup introduces certain measures or restrictions. The experimenter may, for example, randomise the order of the tasks to decrease the chance that certain tasks are complete less often than others. A considerable number of participants is necessary for randomisation to take the desired effect. This sets certain limitations on how the data can be analysed. Hence, to analyse the data we have gathered we used analysis approaches that are currently less common in HCI, psychological, or social science research. Such statistical models often require specialist statistical knowledge and collaboration with experts in the field is recommended. To deal with categorical data and partly incomplete datasets we used a multinomial mixed effect model which was analysed using a Bayesian approach. Using AMT only as a recruitment tool to guide participants to a standard online questionnaire does not suffer from this statistical challenge.

## Conclusions

Overall, the four conditions produced similar distributions for the emotion ratings and we tend to believe that the observed differences, although statistically significant, have little practical implications. Our study shows that MT Batch tasks are an efficient and affordable method for certain types of studies. Although we did not systematically measure the resources necessary to run the different study conditions, our intuitive understanding strongly suggests that the two MT conditions took by far the least effort to setup and run. Beyond that, AMT can be used as a recruitment task alone, which does help HCI researchers to run large numbers of participants in their studies. However, slightly more advanced statistical methods are necessary to analyze MT Batch tasks, due to the fragmented nature of the data.
